# PD-L1 is preferentially expressed on CD44+ tumor-initiating cells in head and neck squamous cell carcinoma

**DOI:** 10.1186/2051-1426-2-S3-P270

**Published:** 2014-11-06

**Authors:** Yunqin Lee, John Sunwoo

**Affiliations:** 1 Stanford University, Stanford, CA, USA

## 

Human tumors consist of heterogeneous populations with different phenotypic characteristics, and these subpopulations can often be distinguished by different surface marker expression. In head and neck squamous cell carcinoma (HNSCC), CD44 has been identified to be a marker of a subpopulation, containing the tumor-initiating cells (or cancer stem cells), which are capable of self-renewal and differentiation into cells lacking this capacity. Significant evidence indicates that these tumor-initiating cells have an immune suppressive phenotype, allowing them to evade the antitumor host immune response, leading some to hypothesize that these cells may contribute to long-term tumor dormancy. In this study, we profiled 21 primary human HNSCC tumors and/or patient-derived xenografts for CD44 expression and PD-L1 (B7-H1) expression by flow cytometry and quantitative RT-PCR. The tumors were categorized into PD-L1-negative and PD-L1-positive tumors. In the PD-L1-positive tumors, we found elevated expression of PD-L1 on CD44+ cells, both at the protein level by flow cytometry and at the transcript level by qRT-PCR. Interestingly, treatment of the tumor cells with recombinant IFN-γ resulted in preferential up-regulation of PD-L1 on CD44+ cells (as compared to the CD44- population) in both PD-L1-negative and PD-L1-positive tumors. To assess the functional significance of the preferential PD-L1 expression on the CD44+ tumor cells, autologous tumor-infiltrating lymphocytes (TILs) were cultured from the primary tumors. By ELISA, autologous co-cultures of CD8+ TILs with sorted subsets of tumor cells showed higher secretion of IFN-γ when incubated with CD44- cells compared to CD44+ cells, suggesting that the CD44+ cells are more immunosuppressive. Blocking the TILs with anti-PD-L1 and anti-PD-1 antibodies partially restored IFN-γ secretion by the TILs incubated with the CD44+ cells, indicating that part of the immunosuppressive properties of CD44+ cells is mediated by the PD-L1-PD-1 signaling pathway. Our results indicate that the preferential PD-L1 expression on tumor-initiating cells may function as one of multiple mechanisms by which long-term tumor-initiating progenitor cells evade the immune response and provide rationale for targeting the PD-1 axis in the adjuvant therapy setting.

